# A web-based carepartner-integrated rehabilitation program for persons with stroke: study protocol for a pilot randomized controlled trial

**DOI:** 10.1186/s40814-019-0439-0

**Published:** 2019-04-25

**Authors:** Sarah Blanton, Patricia C. Clark, Robert H. Lyles, George Cotsonis, Brian D. Jones, Aimee Reiss, Steven L. Wolf, Sandra Dunbar

**Affiliations:** 1 Division of Physical Therapy, Department of Rehabilitation Medicine, Emory University School of Medicine, 1441 Clifton Road NE, Room 213, Atlanta, GA 30322 USA; 2 Byrdine F. Lewis School of Nursing, Georgia State University, Atlanta, GA USA; 3 Department of Biostatistics and Bioinformatics, Emory University Rollins School of Public Health, 1518 Clifton Road, NE, Atlanta, GA 30322 USA; 4 Interactive Media Technology Center, Georgia Institute of Technology, 85 Fifth Street Northwest, Atlanta, GA 30308 USA; 5 Emory University School of Medicine, 1441 Clifton Road NE, Room 206, Atlanta, GA 30322 USA; 6 Atlanta VA Center for Visual and Neurocognitive Rehabilitation, Atlanta VA Health Care System, 1441 Clifton Rd. NE, Room 206, Atlanta, GA 30322 USA; 7 Nell Hodgson Woodruff School of Nursing, Emory University, 1520 Clifton Road, NE, Atlanta, GA 30322 USA

**Keywords:** Caregiver, Stroke, Rehabilitation, Telehealth, Depression

## Abstract

**Background:**

Family carepartner management and support can improve stroke survivor recovery, yet research has placed little emphasis on how to integrate families into the rehabilitation process without increasing negative carepartner outcomes. Our group has developed creative approaches for engaging family carepartners in rehabilitation activities to improve physical and psychosocial health for both the carepartner and stroke survivor. The purpose of this study is to explore a novel, web-based intervention (Carepartner and Constraint-Induced Therapy; CARE-CITE) designed to facilitate positive carepartner involvement during a home-based application of constraint-induced movement therapy (CIMT) for the upper extremity.

**Methods:**

The primary aim of the study is to determine feasibility of CARE-CITE for both stroke survivors and their carepartners. Carepartner mental health, family conflict surrounding stroke recovery, and stroke survivor upper extremity function will be evaluated using an evaluator blinded, two-group experimental design (blocked randomization protocol according to a 2:1 randomization schema) with 32 intervention dyads and 16 control dyads (who will receive CIMT without structured carepartner involvement). CARE-CITE consists of online education modules for the carepartner to review in parallel to the 30-h CIMT that the stroke survivor receives. The intent of CARE-CITE is to enhance the home-based intervention of CIMT, by helping the carepartner support the therapy and create a therapeutic home environment encouraging practice of the weaker arm in functional tasks.

**Discussion:**

The CARE-CITE study is testing the feasibility of a family-integrated rehabilitation approach applied in the home environment, and results will provide the foundation for larger clinical studies. The overall significance of this research plan is to increase the understanding and further development of interventions that may serve as models to promote family involvement in the rehabilitation process.

**Trial registration:**

ClinicalTrials.gov, NCT02703532. Registered 9 March 2016

## Background

Approximately 4.8 million stroke survivors (SS) require assistance from family members who are often untrained and ill prepared for the burdens associated with stroke rehabilitation [1, 2]. Family carepartners (CPs) are key contributors to stroke recovery, but their efforts can lead to a high level of CP burden and depressive symptoms, reduced quality of life (QOL), and increased stress surrounding the recovery process [3–7]. Recent reductions in reimbursement for rehabilitation services and shrinking healthcare resources are creating mounting burdens on the family unit and shifting more of the demand for care to the home [8]. With carepartner well-being affecting health outcomes in both individuals (CP and SS), identifying strategies to effectively support CPs during rehabilitation without contributing to their own burden is a critical need. Creasy et al. [9] evaluated the impact of healthcare provider interactions on CP needs during rehabilitation and found that CPs overwhelmingly emphasized the importance of being involved in treatment planning and expressed expectations to have information and rehabilitation training tailored to family needs [10]. Several studies have shown that improving CP coping and life skills surrounding caring for a chronically ill family member benefits the CP leading to a decrease in measures of depression and caregiver burden and improved QOL [5, 11, 12]. However, to date, a family-focused approach in rehabilitation therapy that addresses CP needs has not been evaluated in stroke research.

Combining findings from rehabilitation therapy and nursing research, we have developed a theory-based intervention—Carepartner and Constraint- Induced Therapy (CARE-CITE)—designed to enhance the therapeutic process by positively engaging CPs during evidence-based SS upper extremity (UE) functional task practice in the home setting [13]. A well-established and structured therapy intervention, constraint-induced movement therapy (CIMT) involves intensive repetitive task practice of the weaker limb combined with restriction of the stronger limb (using a mitt) and has been shown to improve UE function and promote neuroplasticity [14, 15]. While effective, CIMT can also be demanding on the SS and family members [16], frequently requiring additional time and assistance for the SS to complete functional tasks and motivational support from the CP. To address this need, CARE-CITE guides the CP in collaborative goal setting and providing autonomy support (characterized by empathy, choice and reducing use of controlling language) for the SS to promote motivation and creative problem-solving in UE self-management. The theoretical mechanisms of CARE-CITE are rooted in Self-Determination Theory [17] that fosters individual autonomy and confidence resulting in better adherence to self-managed health behaviors. Autonomy-supportive language in task instruction influences motor skill acquisition and self-efficacy in the SS, and is a key ingredient in current motor learning theory in stroke rehabilitation [18]. Although highly applicable to post-stroke motor rehabilitation [18], rehabilitation clinical trials rarely incorporate training designed to support self-management in therapy despite recommendations that this type of behavioral intervention may improve long-term outcomes and enhance motor learning [19].

We initially tested a *workbook version* of the CARE-CITE intervention in the outpatient rehabilitation clinic using a one-group, quasi-experimental design study with 7 CP and SS dyads (SS were > 9 month post-stroke) [20]. SS with mild to moderate UE deficits (ability to initiate active wrist and finger extension) underwent 30 h (10 3-hour sessions) of CIMT while the CP followed an instructional workbook and attended specific CIMT sessions. This initial version of CARE-CITE was feasible as evidenced by high dyad adherence and completion rates (100%) and a high CP-reported value of CARE-CITE in the post-study exit interview. Improvements were seen in CP depressive symptoms and family conflict surrounding stroke recovery and in UE functional tasks for the SS. At exit interviews, CPs rated their participation as worthwhile and believed that their involvement contributed to their SSs’ success. However, they also stated that travel barriers and time demands associated with attending sessions in the clinic were an additional challenge. In response to this feedback, we revised the intervention and developed a digital format of CARE-CITE that can be accessed via a portable electronic device with web-access, such as a tablet or laptop. In collaboration with families, we created exemplary and interactive videos of actual CPs working with SS in activities of daily living to model intervention concepts in a real-world context. Content validity and CP satisfaction with this revised format has been tested to assess feasibility prior to enrollment of the current study [13]. Rehabilitation experts evaluated the website content for accuracy, feasibility, acceptability, problem relevance, and ease of use. CPs (*n* = 6) rated the content for usefulness, ease of use, acceptability, and time to complete on a 5-point Likert-type response scale ranging from 1 = strongly disagree to 5 = strongly agree. Expert reviewers (*n* = 4) rated each module as accurate (4.95), feasible (4.8), easy to use (4.86), acceptable (4.96), and appropriate problem relevance (4.65). On average, all CPs agreed or strongly agreed that the modules were useful (4.42), easy to use (4.6) and acceptable (4.41) and the video content was described as “excellent” and consistently noted as “helpful to understand the information” in each module.

The aim of this paper is to describe the CARE-CITE (NICHD K23 1k23HD080837) pilot study design. The primary objective of this trial is to evaluate feasibility of a web-based, family-focused intervention for stroke survivors and their carepartners. To explore feasibility of CARE-CITE, we will assess participant recruitment and retention, SS and CP adherence to the intervention, CP usability and satisfaction with the CARE-CITE, and occurrence of SS adverse events. The secondary objective of the study is to collect preliminary data indicative of the potential impact of CARE-CITE on CP depressive symptoms, family conflict related to stroke recovery, and SS UE function, with a view toward the design of a larger future trial. To gain insights into potential mechanisms of CARE-CITE, additional preliminary data will be collected on CP self-efficacy, strain, fatigue, general family functioning and SS memory and behavior problems, SS depressive symptoms, and quality of life.

## Methods

Identification and reporting of relevant elements of this protocol are based on the Standard Protocol Items: Recommendations for Intervention Trials (SPIRIT) checklist [21] and Template for Intervention Description and Replication (TIDieR) guidelines for intervention descriptions [22]. This is the first published version of this protocol (November 17, 2018).

### Approvals

Ethical approval was obtained by Emory University Institutional Review Board and this protocol is registered on clinicaltrials.gov (NCT02703532).

### Study Design & Setting

This design is an evaluator blinded, randomized, two group controlled trial with evaluations for the dyad (32 intervention, 16 control) at baseline, immediately post-intervention and at 1-month follow-up. The site for the participant recruitment, screening, evaluations, and personnel training is a stroke research laboratory within a large 56-bed urban rehabilitation hospital located in Atlanta, GA, US. The principal investigator (PI, first author) will perform all participant screening. Licensed physical and occupational therapists will conduct the evaluations and administer the home-based CIMT intervention. Participants will be randomly allocated to either CIMT combined with CARE-CITE intervention or CIMT alone. The web-based CARE-CITE intervention will be accessed on-line by CPs randomized to the intervention group, independent of therapist involvement.

### Recruitment

#### Participants

Forty-eight stroke survivor and carepartner dyads will be recruited for this study. Both CP and SS must be greater than 18 years of age, able to read and write English and able to provide informed consent. Inclusion criteria for the SS include:

- One month to 2 years post-ischemic or hemorrhagic event
- Minimal to moderate upper extremity deficits (ability to initiate wrist and finger extension)
- Presence of CP

Exclusion criteria for SS include:

- Severe cognitive deficits (as indicated by mini-mental test > 24)
- Concurrent participation in other rehabilitation research trials
- Concurrent traditional outpatient therapy for the upper extremity during treatment phase of study
- Major medical issues limiting participation in a rehabilitation program or having another neurological disease like Parkinson’s disease or multiple sclerosis
- Upper extremity pain during functional tasks that may be aggravated by intensive CIMT protocol or limit treatment participation

During the in-person screening phase, participants will be asked about receiving anti-spasticity medications. Oral agents such as dantrolene sodium are allowed, if maintained at consistent dosage. Participants are not allowed to enroll until 3 months after any BOTOX® therapy injections.

CPs will be defined as those individuals who are a spouse/partner or family member dwelling in the same household and self-identified as the primary caregiver of the stroke survivor. Inclusion criteria include willingness to support SS in therapy roles and no significant cognitive deficits as evident by their ability to explain the general purpose of the study and their role as a CP participant to the PI after reviewing the informed consent.

#### Recruitment and retention strategies

Potential participants will be identified by study staff through bi-weekly monitoring of inpatient and outpatient stroke census of the rehabilitation hospital and reviewing electronic medical records for patients that may meet eligibility criteria based on occupational and physical therapy documentation. The primary treating rehabilitation therapists of potentially eligible individuals will be contacted to discuss recommendations for plan of care and appropriateness for inclusion. Additionally, the PI will provide in-services to other regional hospitals and clinics and community stroke support groups and distribute informational brochures. Interested potential participants will be screened for eligibility by the PI and will start the study as soon as recruited. Targeted enrollment will be two dyads per month. To maintain participants in the study, the study PI and study staff will be available to respond promptly to any communication or issue through email and phone and will provide reminder calls and texts to confirm treatment and evaluation appointments. All dyads will receive $100 for study participation.

#### Sample size estimation

Formal sample size calculations are not appropriate for pilot studies [23]. This pilot study will provide important estimates of within-subject and between-subject variability for outcome variables, which will be used to power future full-scale clinical studies. We will focus on the magnitude of the differences for each outcome, consistency of findings, and clinical significance. Julious [24] recommended a sample size of 12 subjects per group as adequate for a pilot study in the context of estimation based on the 95% confidence interval for the mean difference (i.e., the precision about the mean difference as assessed by its 95% confidence interval). He indicated that even a sample size of 12 subjects per group provides useful data about the mean difference and standard deviation for an outcome that is roughly normally distributed. Given this, along with feasibility considerations, we plan to recruit 16 dyads into the CIMT-only control group and 32 dyads into the CARE-CITE group. The larger intervention group will contribute precision to the pilot-based estimates of mean changes and variability for key outcomes, while also improving the lessons to be learned about feasibility to inform future implementation for a larger-scale trial.

#### Randomization

Study participants will be randomized (blocked randomization protocol) according to a 2:1 randomization schema—32 intervention dyads and 16 control dyads (who will receive CIMT without structured carepartner involvement).Using this type of randomization schema will allow for a larger intervention group for assessment of feasibility. Previous studies conducted by this lab have established an expected recruitment rate of approximately 2–3 dyads/month, allowing adequate time to meet target enrollment of 48 dyads during the proposed study time period. This approach offers the opportunity to gather more information on the intervention effects and effect size for future work while still adhering to projected recruitment rates. The blocked randomization protocol will be developed by the study statistician who will create sealed envelopes containing group assignment with sequential dyad numbering (CP1, CP2, etc.) prior to study initiation. Once the dyad completes the baseline evaluation, the sealed envelope will be opened by the PI (unblinding to treatment group allocation), who will inform the CP of allocation.

### Intervention

#### CARE-CITE intervention

Dyads in the experimental group will receive CIMT with CP involvement (CARE-CITE). The CARE-CITE intervention consists of web-based, interactive educational modules (Table 1) for the CP to review during the 4–6-week period of time the SS undergoes ten, 3-h sessions of home-based CIMT. The goal of CARE-CITE is to augment the home-based application of CIMT by guiding the CP in effective strategies that facilitate and encourage the SS to use his/her weaker arm in functional tasks at home. The CPs review the 6 online modules on their own time. The theoretical framework for the intervention is the concept of autonomy support, with text and video that demonstrate ways to encourage empathy (video examples of discussions of CP with SS acknowledging difficulty of task), collaborating on problem-solving (examples of methods to increase or decrease difficulty of activities together), emphasizing importance of offering SS choice in activities to practice (examples of joint goal-setting) and ways to provide non-controlling language (scenarios showing controlling vs. non-controlling language). At the end of each module, the CP is asked to complete 4–6 self-reflection questions that help integrate the educational content into their own lives and activities with the SS. These responses indicate the CP has completed the module and will be sent electronically in real time to the study PI to monitor adherence (see the “Feasibility” section) and progression through the modules and identify any technical problems that may occur.

**Table 1:** Content of the CARE-CITE Intervention for the carepartner

Modules	Content
Overview of Modules Structure	Each module has the purpose, information in text format and video clips to provide additional detail and/or illustrate examples of behavior discussed. At the end of each module are 4–5 reflection questions to allow for application of content and 7 questions to obtain feedback on ease of use, acceptability and usefulness of modules.
Module I: Welcome to CARE-CITE	Description of the CARE-CITE project, overall summary of the modules and intent. Welcome survey for CP to fill out with the research interventionist to practice using website and completing questionnaires. One video clip with introductory information.
Module II: Introduction to Concepts and Applications of CIMT	Overview of CIMT, including behavior contract (CP and individual with stroke determine activities when mitt worn and taken off) and home diary (to record activities/time wearing mitt). Four video clips with information about wearing the mitt, behavior contract, home diary, and committing to practice.
Module III: Practice and Goal Setting	Review of role of practice in driving neuroplasticity and recovery after stroke. Discussion of challenge threshold, making mistakes while learning new skills. Guidance provided for problem solving to adapt functional activities at home, both reducing complexity when a task was too difficult and increasing challenge when the task was easily mastered. Two to three brief video clips for each of six themes of practice.
Module IV: Autonomy Support – Creating Partnerships	Creating an Autonomy-Supportive Environment – How to be empathic, problem solve in performing tasks in the home setting, use non-controlling language and offer choice. Examples include suggesting CP wear mitt on non-dominant hand while trying tasks and offering alternative activities when individual with stroke becomes frustrated during a challenging task. Recognizing challenges and exploring ways to improve communication (avoid controlling language such as “you should exercise”, “you have to do this”). Seven video clips illustrating understanding another’s viewpoint, problem-solving strategies, providing rationale and providing choice.
Module V: Taking Care of Yourself as a Carepartner	CP self-care – recognizing demands of caregiving role, strategies for stress reduction, opportunities for self-care activities and community resources. (no videos)
Module VI: Reflections	Three videos (limited text) of stroke survivors reflecting on rehabilitation and recovery. Encouraging CP reflection on his/her role in recovery of the individual with stroke.

Table reprinted by permission of the publisher (Taylor & Fancis Ltd., http://www.tandfonline.com) from [13]

For both groups, the CIMT sessions focus on functional task practice of the upper extremity. The SS chooses tasks and the practice session schedule. During the first CIMT session, the SS will be introduced to the approach of CIMT, and how and when to safely use the mitt on the stronger hand. This information will be conveyed through a behavioral contract that will document specific activities for which the mitt should (and should not) be worn. The SS will be encouraged to wear the mitt as much as possible during waking hours with a goal of 5 h/day during the entire treatment period. To document activities attempted with the weaker hand and create opportunities for collaborative problem-solving with the therapist, the SS completes a home diary. In addition to providing information regarding SS adherence to wearing the mitt, the diary offers an opportunity for the SS and therapist to identify and discuss challenges and difficulties that might be experienced outside of therapy sessions. Graded and progressive task practice will address overall upper extremity function, strength, range of motion, and fine motor dexterity. Recommendations for functional activity practice between sessions to address SS movement impairments will be based on the SS (and CP in CARE-CITE group) goals, (e.g., to increase functional grasp, reaching and release, practice loading and unloading dishwasher, progressing to heavier items as tolerated). Each therapy session will begin with check of vitals, and questions assessing any pain, fatigue, and incidence of falls or medical visits. The therapist will review the home diary and discuss any successes or challenges encountered since the last visit. The SS will then be asked to identify tasks to be practiced during the session and order of practice. After the completion of the final CIMT session the mitt use will be discontinued, but the SS will be encouraged to continue progressing with functional tasks and goals.

#### Control group

In the control group, the SS will receive the same CIMT intervention as the experimental group. Although the CP may be in the home when the stroke survivor receives therapy, the CP will not receive any materials about how to engage with the SS other than review of safety considerations when the SS is wearing the mitt.

#### Standardization

For the CARE-CITE intervention group, the study PI (primary author) will instruct all CPs in the use of the CARE-CITE modules, monitor adherence to intervention, and download data from module reflection and feedback questions. CIMT will be delivered by licensed occupational and physical therapists who will not have access to the CP modules/CARE-CITE content. The study PI will monitor training, standardization, and delivery of CIMT through weekly check-ins with each therapist via email and phone communication and will train and standardize the study evaluator. All efforts will be made to use one evaluator for all study evaluations (primarily within each dyad) to reduce variability. Data collection forms will be standardized for evaluators and intervention therapists to facilitate protocol adherence.

### Outcomes

Data collection for SS and CP will be completed at a rehabilitation hospital stroke laboratory in separate rooms by the evaluator, blinded to group assignment, study hypotheses, and CARE-CITE intervention. Outcomes will be measured at baseline, within 1 week of completion of CIMT treatment and at 1 month follow-up.

#### Outcomes for Carepartner and stroke survivor

Primary outcome measures for the CP are depressive symptoms and family conflict surrounding stroke recovery and upper extremity function for the SS. These measures have been tested in this population previously. Additional exploratory measures will be collected to gain insights into possible mechanisms of CARE-CITE and include CP self-efficacy, strain, fatigue, well-being related to caregiving, general family functioning and stroke survivor’s memory and behavior problems, depressive symptoms, UE self-efficacy, and quality of life. Table 2 lists primary outcome measures, process variables and usability measures, descriptions, and established reliability and validity.

**Table 2:** Outcome measures collected at baseline, post-intervention, and 1 month follow-up

Variable	Measures	Description	Reliability/validity
Primary outcomes			
CP depression symptoms	CES-D	20-item, Likert-type scale	Established validity, internal consistency, reliability [25, 26]
CP family conflict	Family Caregiver Conflict Scale (FCCS) about Stroke Recovery	15-item, Likert-type scale higher scores/higher conflict	Established content/construct validity in stroke CP; reliability Cronbach’s alpha of .93 [27]
SS upper extremity function	Wolf Motor Function Test (WMFT) Motor Activity Log (MAL)	15-item speed measures;2-item strength; low score/faster speed 30-item questionnaire, Likert-type scale; high score/high quality UE use	Inter-rater reliability *r* = .97; valid in the stroke population [28]
Process variables			
CP/SS autonomy support environment	Family Care Climate Questionnaire FCCQ-CP/FCCQ-SS	14-item, Likert-type scale. Higher scores/higher autonomy support perception	Internal consistency > .70; Construct validity supported- higher FCCQ-SS scores related to SS lower perception of criticism, higher family emotional involvement-higher satisfaction with family support (*p* ≤ .05) [29]
CP fatigue	Piper Fatigue Scale	22-item scale, Likert-type scale. Higher score/higher fatigue	Strong internal consistency reliability Cronbach’s alpha of .97 and construct validity in stroke carepartners [30, 31]
CP strain	Carer Strain Index -CSI (modified)	13-item questionnaire, binary yes/no; higher score/higher strain	Good reproducibility and validity in stroke carepartners, Cronbach’s alpha of .83 [32–35]
CP well-being related to caregiving	Bakas Caregiving Outcome Scale (BCOS)	15-items; 7-point scale; higher scores/more positive caregiving outcomes	Satisfactory reliability and validity in stroke carepartner, Cronbach’s alpha of .90 [36]
CP family functioning	Family Assessment Device (FAD)	27-items; Likert-type scale. Higher score/unhealthy functioning	Concurrent and predictive validity, internal consistency reliability, sensitivity and specificity demonstrated various samples [37] and used in stroke studies [38, 39]
CP perspective of SS memory and problem behaviors	Memory & Behavior Problems Checklist (MBPC)	19-item scale; Likert-type scale. Higher score/higher frequency	Reliability and validity established in dementia population, and internal consistency reliability coefficient = .73 in stroke [40, 41]
SS quality of life	Stroke Impact Scale (SIS)	59-items, 8 domains function	Test-retest reliability ICC = 0.70 to 0.92; Internal consistency alpha coefficient of 0.83–0.90 [42]
SS UE self-efficacy	Confidence in Hand and Movement Scale (CAHM)	20-item (scale 0–100)UE confidence for functional tasks; high scores/high confidence	Reliable and valid with moderate relationship with WMFT 3–9 months post-stroke [43]
SS upper extremity function	Upper Extremity Fugl-Meyer	33-item, 3-point ordinal scale; higher score/higher function	Established reliability and validity in stroke popul. [44, 45]
CARE-CITE usability			
CP experience in CARE-CITE	Exit Interview	Three sections assessing confidence in care, value of participation and aspects of CARE-CITe	Interview guide will be reviewed by content and qualitative experts prior to use.
CP satisfaction with CARE-CITE	Feedback forms at end of CARE-CITE modules	5-items, Likert-type scale; higher scores/higher satisfaction	Interview guide will be reviewed by content and qualitative experts prior to use.
CP confidence in using technology	Modified Computer Self Efficacy Scale (MCSES)	10-item, Likert-type scale. Higher scores/higher self-efficacy	Established reliability and validity [46].
CP experience using CARE-CITE	Post-Study System Usability Questionnaire (PSSUQ)	19-item, Likert-type scale. Lower scores/greater usability of instrument	Established reliability and validity [47]

#### Additional assessments

Participant characteristics will be gathered from medical records (for SS) and baseline information questionnaires (SS and CP). For both CP and SS, information includes demographics (age, gender), marital status, education level, income, work status, any changes in work since stroke, co-morbidities, and current medications. Specific information for the SS includes type of stroke, time post-stroke, and hand dominance and for CP, relationship to SS and status of serving as caregiver for other members of family beside SS. Because the CARE-CITE intervention is web-based, we will also assess at baseline the CP’s confidence in using technology.

#### Feasibility

To assess feasibility of CARE-CITE, we will assess participant recruitment and retention, SS and CP adherence to the intervention, occurrence of SS adverse events, and CP perceptions of usability and satisfaction with CARE-CITE**.** Recruitment rates will be calculated based on the percentage of those participants enrolled and randomized from those screened. Recruitment will be deemed feasible if the target enrollment of 48 dyads (2–3 dyads per month) is reached during the study timeframe. Retention of participants will be tracked, recording the percentage of dropouts from each group. An acceptable retention rate will be 85% of enrolled participants for completion of post-evaluation (80% for one-month follow-up).

Adherence to intervention for SS will be measured by number of CIMT sessions attended (10 total sessions) and total hours (30 h) of training completed. Criteria for adherence completion of CIMT will be a minimum of 27 h. Minimal percentage of target adherence rate for feasibility is 85% of enrolled SS completing CIMT. Review of the SS home diary will provide data on daily and overall mitt wearing during the treatment as well as functional activities attempted. CP adherence will be measured by number of modules reviewed (6 total modules) as indicated by questions completed at the end of each section. Criteria for adherence completion of CARE-CITE will be a minimum completion of 5 of the 6 modules. Minimal percentage of target adherence rate for feasibility is 85% of CP in the CARE-CITE group completing modules. Based on work by Bakas and colleagues [48], adapted questions will be used to evaluate CP usability and satisfaction of CARE-CITE. At the end of each of the six modules, CP will be instructed to complete these questions immediately after reviewing a module. Satisfaction will be defined as (1) usefulness of overall content, (2) usefulness of written text, (3) usefulness of videos, (4) ease of use, and (5) acceptability. Each area will be rated using a 5-point Likert type response scale ranging from *1* = *strongly disagree* to *5* = *strongly agree* and average scores calculated for each subscale as well as a total score. CP will record time (minutes) to complete each module and these averages will be summed to obtain total time to review all modules. To supplement this data, one open-ended question will be used for general comments or improvement suggestions. The Post-Study System Usability Questionnaire [47] will be administered to experimental group CPs at the post-treatment evaluation to gather additional data on CARE-CITE usability [46]. At the 1-month post-evaluation, the CP’s perceptions of the intervention will be gathered through a three-section CP exit interview administered by the study PI in a private room. Section A has 15 items, with a response scale of 0–100 (0—very uncertain to 100—very certain), and addresses confidence in care, with sample questions such as “How confident are you that you can encourage your loved-one when she/he is frustrated with a task?” Section B has 5 items about value of participation in the study, such as “Given the time commitment and effort for you to take part in the education project and its effect on your ability to help your loved one, how worthwhile has the participation in the education project been to you personally?” with a response scale of 1–7 (i.e., 1—not worthwhile to 7—very worthwhile) and two open-ended questions. Section C assesses aspects of helpfulness for the intervention such as “Given your experience with the education project, how helpful do you feel each of the following aspects was for achieving results in your particular case?” (Being able to view intervention from your home; homework activities between sessions, format of using CARE-CITE website) with a response scale of 1–7 (i.e., 1—not helpful at all to 7—very helpful). CPs will be asked to provide additional feedback about helpful areas of the intervention and areas for improvement through two additional open-ended questions. Modeled after others used in research [43], this investigator-developed exit interview was reviewed by experts in caregiving and stroke for content validity.

### Harms

For the SS, adverse events will be monitored at each home-based treatment session by intervention therapists delivering the CIMT and they will report to the study PI if any adverse event occurs. Although no significant risks have been associated with the administration of upper limb task practice in the home, the primary risks involve fatigue or frustration when attempting challenging tasks with the weaker limb and falls in those individuals with balance impairments. At each training session, the therapist will ask the SS questions related to levels of pain, fatigue, and any incidence of falls or medical appointments since the previous session. A behavior contract will be reviewed with the patient to identify any tasks in which safety is a concern or that should be avoided when the carepartner is not present. These guidelines are individually tailored to the participant and the carepartner is involved in the process. Risks to CPs are minimal because this intervention is psychoeducational. Because we will be administering a questionnaire to CPs that has a cut-off score that indicates possible depression, we will notify CPs who score above the established cut-off of 16 on the CES-D that their response indicates they may need an evaluation for depression. We will recommend that they contact their primary health care provider for a referral. If they do not know of someone, we will provide resources for their consideration. The consent form will contain a statement explaining this process. All adverse events will be reported, documented, and categorized as serious (death, life-threatening or related inpatient hospitalization) or non-serious; anticipated or unanticipated; related, potentially related or non-related to the intervention as defined by the local institutional review board. Date of onset, action taken, outcome, and any changes in procedures/treatment will be noted. Due to the low risk involved in the study for CP and SS, there will be no Data Safety and Monitoring Board, and all serious adverse events will be reviewed by the study team (SB, PC, SD) to determine whether safety problems would warrant termination of the trial.

### Study procedures

**Fig. 1:**
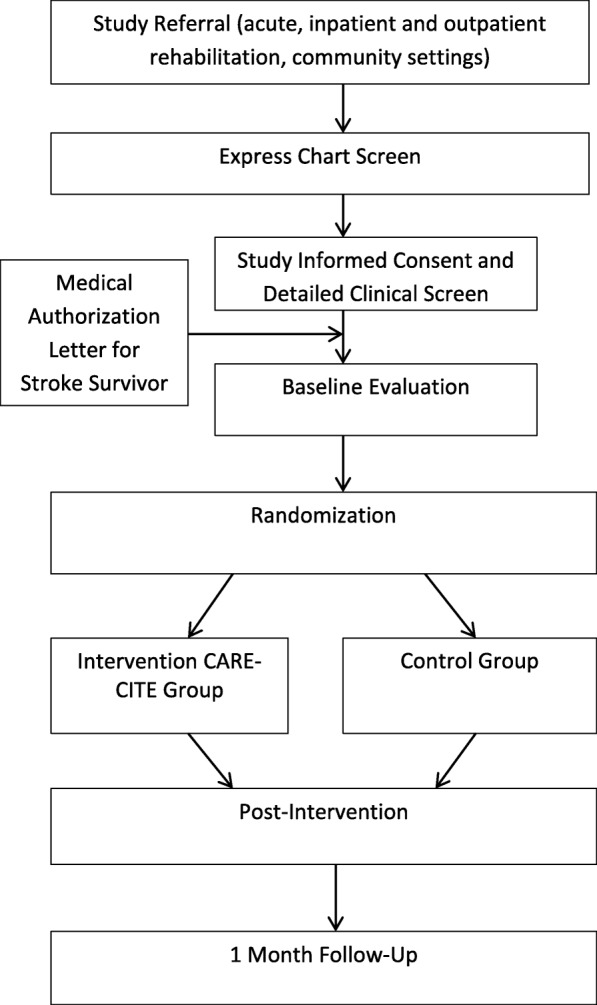
Consort flow chart: evaluation of a carepartner-integrated telehealth rehabilitation program for ersons with Stroke (CARE-CITE)

Figure 1 depicts the study design. Before study participation, participant eligibility will be confirmed via phone screen, chart review and initial clinical evaluation and physician consent. Enrollment will be defined as the review and signing of the informed consent document by the participant and study PI, just prior to baseline evaluation. Immediately following baseline testing, study participants will be randomized by the PI, who will inform the CP of allocation and provide information regarding accessing CIMT modules for the intervention group.

SSs will start the home-based CIMT within a week of randomization and continue for 4–6 weeks until full dosage (total of 30 h) of CIMT is administered. Outcomes will be measured at baseline, within 1 week of completion of CIMT treatment (post-test) and 1 month (after post-test) follow-up.

### Data management and analysis

All quantitative data storage will be maintained through REDCap electronic database. REDCap (Research Electronic Data Capture) [49] is a secure (compliant with US healthcare confidentiality legislation requirements), web-based application designed to support data capture for research studies, providing (1) an intuitive interface for validated data entry, (2) audit trails for tracking data manipulation and export procedures, (3) automated export procedures for seamless data downloads to common statistical packages, and (4) procedures for importing data from external sources. REDCap data entry is completed by trained research assistants. To minimize data entry errors, all data are double checked for verification by a second research assistant.

Standard data cleaning, identification of missing data, and internal consistency reliability for standardized scales will be completed. All quantitative statistical analyses will be performed using SAS statistics for Windows (Version 9.4). Descriptive statistics (e.g., frequencies, means, ranges, standard deviations) will be examined for all relevant variables for summarization, as well as to identify unusual or suspect values requiring review and confirmation. Initial data analysis will also include descriptive statistics on feasibility measures of recruitment rate, retention, CP usability, and satisfaction with CARE-CITE (described under the “Feasibility” section). To aid in establishing estimates of variability useful for postulating effect sizes when designing larger follow-up studies, mean and median changes from baseline to 1 month and standard errors of the means within each group (control and CARE-CITE intervention) will be examined and reported. Confidence intervals for the difference in mean changes for major study variables between the CARE-CITE intervention and control groups will be reported as descriptive statistics in order to provide preliminary information about possible intervention effects based on the pilot study. These major study variables include carepartner depression (CES-D), family conflict surrounding stroke recovery (FCCS), autonomy support (FCCQ-P and FCCQ-F), and stroke survivor upper extremity function (WMFT and MAL). Estimates of inter-correlations among the study variables, including dyadic variables that could influence response to the intervention, will be summarized as additional information potentially helpful toward the design of future studies. The biological variables of CP age, sex, gender and relationship of CP to SS (e.g., spouse, adult child) and other participant characteristics such as SS other health problems will be examined and potential relationships explored to gain insight into possible confounding factors.

### Auditing

Authorized representatives of NIH, regulatory agencies, and the Institutional Review Board will be permitted to review all research records to monitor study safety, progress, and procedures for quality assurance at any time.

### Dissemination

Results of the study will be shared through peer-reviewed manuscripts and conference presentations. The proposed dissemination plan will be refined through meetings with study leadership, and authorship guidelines will be reviewed with all study personnel. The PI will arrange follow-up presentations with community stroke groups and therapy clinics involved in study recruitment communications.

## Discussion

While evidence supports the importance of addressing CP needs, no studies have systematically integrated a CP intervention (directed toward promoting an autonomy-supportive environment) with a rehabilitation therapy approach. This protocol details the methodology to evaluate the CARE-CITE intervention designed to enhance the therapeutic process by facilitating CP involvement and providing education about stroke recovery. The results of this pilot RCT will inform the development of a larger clinical trial to test the efficacy of CARE-CITE by gaining preliminary estimates of efficacy and components of variability associated with CARE-CITE in chronic stroke. Additionally, information from this study will assist in determining appropriateness of outcome measures, evaluating treatment adherence for SS and CP, monitoring incidence of severe adverse events, measuring recruitment rates, and gaining information to inform power calculations for the RCT sample size. Potential limitations may be encountered. For example, while CP of any sex/gender will be recruited, most CPs are female and this may limit interpretations of any sex/gender differences found.

This family-centered intervention is a key advancement, moving the rehabilitation field toward stroke care that effectively addresses both survivor and CP needs in tandem. Results of this area of research have the potential to guide the integration of similar CP interventions with other therapy approaches.

## Trial status

This trial was registered on clinicaltrials.gov (NCT02703532) on March 9, 2016. Recruitment of participants was initiated on March 24, 2016. To date, we have recruited 67% of the participants.

## Abbreviations

BCOS: Bakas Caregiving Outcomes Scale
CARE-CITE: Carepartner and Constraint-Induced Therapy
CES-D: Center for Epidemiological Studies-Depression scale
CIMT: Constraint-Induced Movement Therapy
CP: Carepartner
CSI: Caregiver Strain Index
FAD: Family assessment device
FCCQ: Family Care Climate Questionnaire
FCCS: Family Caregiver Conflict Scale
MAL: Motor activity log
MBPC: Memory and Behavior Checklist
MCSES: Modified computer self-efficacy scale
PSSUQ: Post-Study System Usability Questionnaire
SIS: Stroke Impact Scale
SS: Stroke survivor
UE: Upper extremity
WMFT: Wolf Motor Function Test
